# From WSN towards WoT: Open API Scheme Based on oneM2M Platforms

**DOI:** 10.3390/s16101645

**Published:** 2016-10-06

**Authors:** Jaeho Kim, Sung-Chan Choi, Il-Yeup Ahn, Nak-Myoung Sung, Jaeseok Yun

**Affiliations:** 1 School of Electrical and Electronic Engineering, College of Engineering, Yonsei University, Seoul 03722, Korea; jaehokim@yonsei.ac.kr; 2 IoT Platform Research Center, Korea Electronics Technology Institute, Seongnam 13509, Korea; csc@keti.re.kr (S.-C.C.); iyahn@keti.re.kr (I.-Y.A.); nmsung@keti.re.kr (N.-M.S.); 3 Dept of Internet of Things, SCH Media Labs, Soonchunhyang University, Asan 31538, Korea

**Keywords:** wireless sensor network, Internet of Things, Web of Things, oneM2M, open API

## Abstract

Conventional computing systems have been able to be integrated into daily objects and connected to each other due to advances in computing and network technologies, such as wireless sensor networks (WSNs), forming a global network infrastructure, called the Internet of Things (IoT). To support the interconnection and interoperability between heterogeneous IoT systems, the availability of standardized, open application programming interfaces (APIs) is one of the key features of common software platforms for IoT devices, gateways, and servers. In this paper, we present a standardized way of extending previously-existing WSNs towards IoT systems, building the world of the Web of Things (WoT). Based on the oneM2M software platforms developed in the previous project, we introduce a well-designed open API scheme and device-specific thing adaptation software (TAS) enabling WSN elements, such as a wireless sensor node, to be accessed in a standardized way on a global scale. Three pilot services are implemented (i.e., a WiFi-enabled smart flowerpot, voice-based control for ZigBee-connected home appliances, and WiFi-connected AR.Drone control) to demonstrate the practical usability of the open API scheme and TAS modules. Full details on the method of integrating WSN elements into three example systems are described at the programming code level, which is expected to help future researchers in integrating their WSN systems in IoT platforms, such as oneM2M. We hope that the flexibly-deployable, easily-reusable common open API scheme and TAS-based integration method working with the oneM2M platforms will help the conventional WSNs in diverse industries evolve into the emerging WoT solutions.

## 1. Introduction

Wireless sensor networks (WSNs) are multi-functional components integrated with embedded computing systems in order to observe the status of surrounding environments, but also control functions of mechanical and electrical systems in a diverse range of industries. Over the past decade, they have been widely used in most application domains, including consumer electronics, transportation, factory, energy, automotive, agriculture, entertainment, aerospace, and so on. One example of WSNs is the home area network inside smart homes that is responsible for monitoring and controlling various functions of home appliances.

Due to technological advances in contactless data exchange (RFID and NFC), distributed sensor networks, short-range wireless communication (ZigBee and Bluetooth), and universal mobile access (cellular networks and WiFi hotspots), things in the world (most of them rely on WSNs) can be connected with each other, building a global network infrastructure, called the Internet of Things (IoT). The term IoT was originally coined by Ashton based on RFID technology [1], but it today represents a technological revolution in which the physical and virtual things in the world can communicate with each other and share their status and surroundings, enabling them to work together cooperatively to perform daily tasks without explicit human intervention [2].

**Figure 1:**
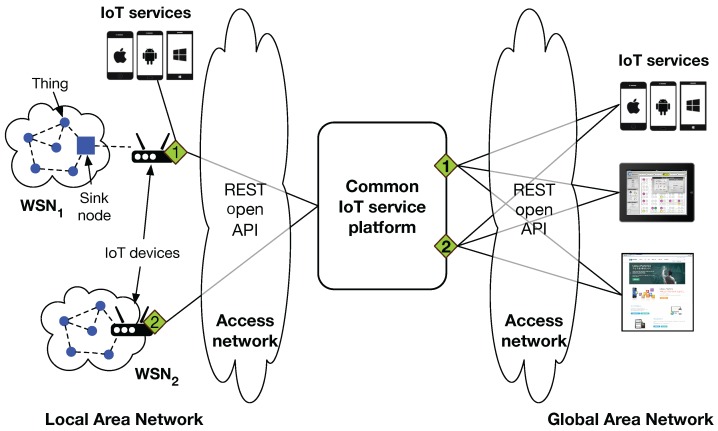
Requirements of providing the common IoT service platform and REST open API to make it easy to integrate legacy (marked as WSN_1_) and new (marked as WSN_2_) WSN-based systems into other IoT systems for building IoT application services.

As explained above, WSNs have a potential to become a building block for IoT systems with the help of Internet connectivity. However, since most WSNs are implemented based on a wide range of hardware systems and diverse network configurations, the heterogeneity problem needs to be addressed with a common, standard service platform that can mask the complex underlying hardware and network systems, while providing a set of useful application programming interfaces (APIs) to applications. In particular, with advances in Web technologies, open APIs based on the representational state transfer (REST) architecture are widely adopted in many Internet-based services and applications. Figure 1 illustrates the requirements of the common IoT service platform and REST open API for building IoT applications being connected with WSN systems. WSN_1_ represents a legacy system whose sink node (or legacy gateway) is linked with an IoT device having Internet connectivity while WSN_2_ represents an IoT-enabled WSN system *per se*. Both are connected to the common service platform so that IoT applications can leverage all of the data and functions of WSNs via REST open APIs (i.e., green diamonds in Figure 1), regardless of the type of WSNs. Furthermore, a suite of well-organized open APIs (e.g., using the same API preceded with different base addresses according to local and global area networks) can help developers build software applications effectively, becoming a basis for building and maintaining IoT systems based on a variety of WSNs. Accordingly, standard IoT platforms and well-defined open APIs will be necessarily required for existing or future WSN systems to be flawlessly evolved as a part of IoT systems.

In this paper, we present a Web-based, open API-driven IoT service framework for WSN systems based on standard IoT software platforms, called Mobius and &Cube, available to IoT service servers and IoT devices, respectively. Both IoT platforms are designed to be compliant with a globally-applicable IoT standard, oneM2M, enabling them to be interconnected with other oneM2M standard platforms. With an introduction of a thing-specific layer for IoT devices, called the thing adaptation layer (TAL), we can provide a systematic way of creating REST open APIs with which application developers will develop application software to interact with the things directly wired or connected via WSNs to IoT devices. Finally, we demonstrate three pilot IoT application services based on a set of ZigBee and WiFi devices connected with the IoT platforms.

Our work is particularly interesting because it is the first effort based on oneM2M-compliant platforms to extend legacy systems, such as WSN systems, into the emerging IoT world, in particular on a global scale via REST APIs. The implemented prototype services demonstrate well the flexibility and reusability of the open API scheme based on the oneM2M standards and object identifier-based IDs for IoT devices. We therefore expect that the proposed method will be able to help a myriad of WSN systems deployed across multiple domains to be incorporated into the future IoT services in a standardized way.

The remaining organization of the paper is as follows. Section 2 presents open APIs in various IoT platforms and products. Section 3 introduces oneM2M standards and our oneM2M-compliant platforms, Mobius and &Cube, as well as thing (i.e., WSN elements) adaptation techniques for exposing open APIs through the platforms. In Section 4, we demonstrate how the proposed open API scheme increases the flexibility and reusability of the open APIs in IoT service development. Section 5 illustrates three IoT services, including Planty, iThing, and iDrone, to show the practical usability of our platforms and common open APIs. In Section 6, we discuss several limitations and remaining challenges, and Section 7 offers concluding remarks.

## 2. Related Works

Several literature works presented the necessity of providing standardized open APIs in a network platform. Grønbæk proposed the architecture for the IoT systems and APIs that can decouple service development from the underlying network and protocols [3]. Mulligan et al. summarized the activities of Google’s Open APIs and platforms (i.e., OpenSocial and Android) for the developer community and highlighted the direction of open APIs for the next-generation networks (NGNs) and their standardization [4,5]. Guinard et al. described the Web of Things (WoT) architecture and best-practices based on the REST APIs [6]. Chung et al. presented a social WoT platform and lightweight smart home gateway for IoT application development [7,8]. Similarly, Corredor et al. presented a lightweight WoT open platform for healthcare services [9]. Pérez et al. introduced the COMPOSE API that allows things, users, and platforms to communicate [10]. REST API-based IoT platforms have also been studied for big data and environmental monitoring [11,12].

Castro et al. presented a survey of M2M/IoT platforms and standard-based open API connectivity [13], including Sen.se [14], EVRYTHNG [15], AMEE [16], RunMyProcess [17], Axeda [18], and ThingWorx [19]. Sneps-Sneppe and Namiot introduced the progress of open APIs for M2M applications in the ETSI standards and proposed a framework for client-side service discovery and inter-application communication for M2M systems to be able to find appropriate services based on the user’s preference [20,21]. Fraunhofer FOKUS’s team developed its own M2M platform (called OpenMTC) together with a set of REST APIs that enable third party applications to access the platform [22]. Recently, they introduced standard M2M APIs for the cloud-based Teclo service platform [23] and extended the OpenMTC architecture to support a software development kit (SDK) that enables third party developers to create their own M2M applications [24]. Liu et al. proposed an efficient naming, addressing, and profile services in IoT environments with a middleware platform, called NAPS, and a unique device naming and addressing convention [25].

Besides the telecommunication standard organizations, such as ETSI, there has been efforts to build IoT standards for supporting service scenarios based on IoT software platforms and open APIs. In 2012, the oneM2M initiative was founded to create global standards for a common IoT/M2M service layer, which can be readily embedded within various hardware and software to connect the myriad of devices in the field with IoT/M2M application servers worldwide [26]. The oneM2M developed and published the standard open APIs with the REST approach for simplifying development phases for IoT/M2M applications.

In the emerging IoT market, more and more companies have presented innovative IoT products and REST API-based service and development frameworks. First, the Nest Thermostat is a well-known IoT product, which can learn the user’s activity patterns and then automatically control HVAC systems to save energy [27]. It also supports REST APIs for interacting with the Nest Lab’s products, enabling developers to create their own applications. Another compelling example is Philips Hue, an LED light bulb embedded with a ZigBee transceiver that can be controlled via smartphone applications [28]. In 2013, Philips released its open APIs and SDK for iOS developers (now also available for Android), as well as guides for both hardware and software makers.

As described above, the open API is considered as a key enabling factor and accelerator for building IoT systems. Furthermore, the REST API is mainly considered as their open API because of some advantages, such as supporting lightness for devices with limited capabilities (e.g., WSNs), creating loosely-coupled services and being easily applicable for the resource-oriented architecture (ROA). However, we need to consider additional requirements for the REST open API that can support IoT-inspired environments consisting of a myriad of IoT devices, gateways, and servers. First, the open APIs for IoT systems need to support a flexible API structure allowing for various service scenarios and diverse network configurations. In contrast to conventional Web services where the APIs for accessing resources are exposed only through the Web servers, open APIs for IoT systems need to account for various service scenarios where the resource of interest could be located in a particular device, as well as in the corresponding gateway or server as virtual entities. Thus, the open APIs need to support developers with a flexible open API structure to help them easily and rapidly create application software suitable for various service scenarios. Second, we need to consider the reusability of open APIs for efficiently dealing with the access request to a common resource in a large number of same-type IoT devices. Hundreds of thousands of same-type devices would be deployed in the IoT environment, but we could help developers create a service application that could be applied to all of the devices using a commonly-structured open API.

## 3. oneM2M IoT Platforms

Without standards, IoT solutions would be developed independently for different vertical domains, causing high fragmentation problems and increasing the overall cost for development and maintenance. In order to mitigate the highly-fragmented IoT ecosystem, several standardization bodies and commercial alliances have come together and published standard specifications on IoT systems. Among them, we focus on oneM2M standards and create software platforms for devices and gateways (i.e., &Cube) and servers (i.e., Mobius) consisting of IoT systems. Open sources for the oneM2M platforms are available in the OCEAN (Open allianCE for IoT stANdards), an open source-based global partnership project for IoT [29].

### 3.1. oneM2M Standards Overview

The oneM2M global initiative has made an effort to standardize a common service layer platform for globally-applicable and access-independent M2M/IoT services. Swetina et al. summarized well the oneM2M standardization activities [30]. The oneM2M first collected various compelling use cases from a wide range of vertical business domains. After that, it formulated requirements for the oneM2M common service layer [31] and then designed the system architecture [32].

**Figure 2:**
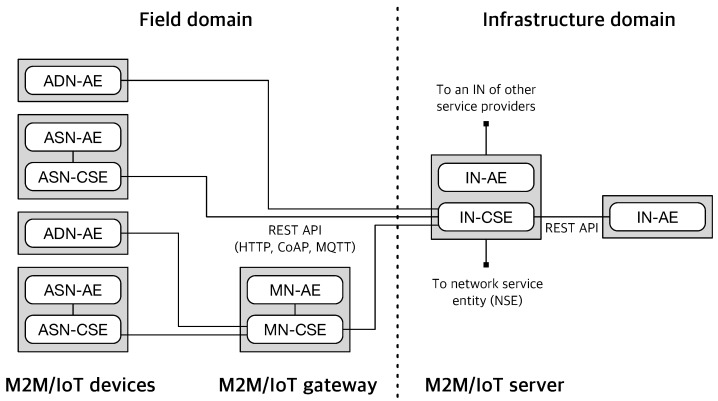
oneM2M reference architecture.

Figure 2 presents the oneM2M reference architecture model. Considering a configuration scenario when oneM2M systems are deployed, the oneM2M architecture divides M2M/IoT environments into two domains (infrastructure and field domain) and defines four types of nodes, which reside in each domain: infrastructure node (IN), middle node (MN), application service node (ASN), and application dedicated node (ADN). Furthermore, the oneM2M architecture is based on a layered model, which comprises the application layer, the common service layer, and the underlying network service layer, each of which is represented as an entity in the oneM2M system [32,33]. The application entity (AE) represents application services located in a device, gateway, or server. The common service entity (CSE) stands for an instantiation of a set of common service functions (CSFs) with which the oneM2M platform provides common services for the M2M/IoT service environments; for example, device registration and data management (see Figure 5 for all 12 functions). The network service entity (NSE) implies network services from the underlying network to be utilized by the CSEs.

**Figure 3:**
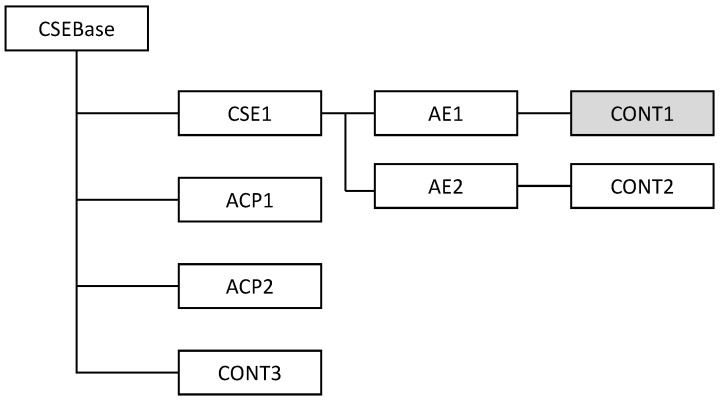
Resource-oriented architecture (ROA)-based oneM2M resource structure.

The oneM2M architecture adopted the ROA model, and thus, the services and data that oneM2M system supports are managed and exposed as a resource information model. With the ROA concept, resources in the ROA can be uniquely addressed by the uniform resource identifier (URI), and the interactions with the resources are supported by the basic four CRUD (create, retrieve, update, and delete) operations. Therefore, based on the URI and CRUD operations, APIs have a simple structure similar to the Web APIs. As shown in Figure 3, oneM2M system manages its resources as a hierarchical structure. Starting from the root of CSEBase, resources are created as child resources, which represent services and data in the oneM2M system. When accessing the resource, the address of the resource should be represented as a hierarchical address that looks like the resource structure. For example, considering the CONT1 resource as shown in Figure 3, its address with which it can be accessed is CSEBase/CSE1/AE1/CONT1. Additionally, oneM2M specifies a service layer protocol, such as primitives (common service layer message format) [34], as well as its protocol binding with the underlying delivery protocol including HTTP, constrained application protocol (CoAP) and message queue telemetry transport (MQTT) [35,36,37]. The oneM2M standards also consider security aspects [38] and device abstraction and management technologies, like open mobile alliance (OMA) device management (DM) and broadband forum (BBF) TR-069 [39,40].

**Figure 4:**
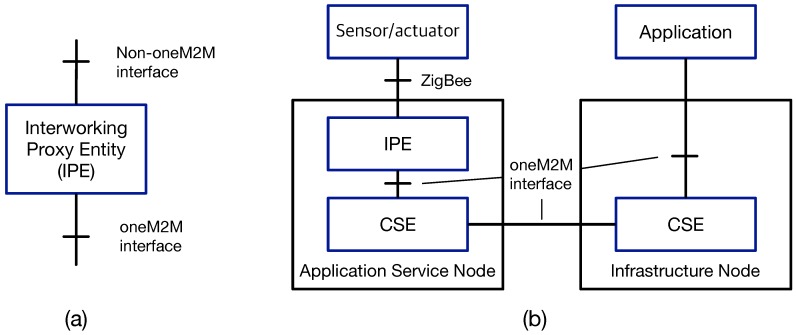
(**a**) Interworking proxy entity (IPE) for interworking with non-oneM2M systems and (**b**) an interworking example for ZigBee-based systems.

The oneM2M standards are now developed for creating globally-applicable, access independent IoT/M2M applications, but there exists a huge number of non-oneM2M systems already deployed across multiple domains. Accordingly, for interworking with the non-oneM2M systems, oneM2M specifications define a specialized AE, called the interworking proxy entity (IPE). Figure 4 illustrates the concept of IPE and an example of building an oneM2M-based application for monitoring non-oneM2M systems (i.e., ZigBee devices). IPEs are mainly characterized by two features [32]: providing non-oneM2M reference points (e.g., ZigBee) and remapping the related data model into the oneM2M-defined data model, which are eventually exposed to other oneM2M systems (e.g., the monitoring application wired to the IN-CSE in Figure 4). When translating data models, a full semantic interworking between two data models would be possible with the help of the related protocol interworking, but otherwise, the encoded non-oneM2M data and command will be packed into a list of oneM2M containers. Consequently, the oneM2M applications need to know the protocol rules of the non-oneM2M systems to decode and understand the content within the containers.

### 3.2. Mobius and &Cube

**Figure 5:**
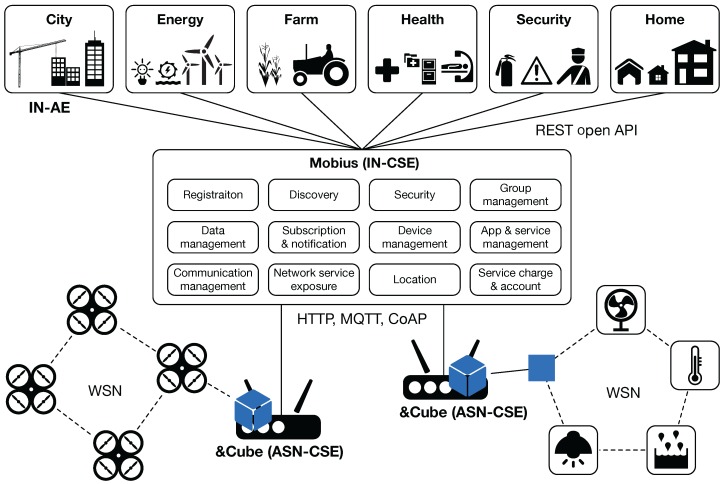
A block diagram of overall architecture and interface for an IoT system composed of our oneM2M standards-compliant platforms (Mobius and &Cube).

Mobius is a common IoT service platform (i.e., IN-CSE) located in the infrastructure domain, whereas &Cube is a software platform for IoT devices located in the field domain (i.e., MN, ASN, ADN). In the previously-published papers, we have presented the full details on Mobius [41] and &Cube [42], respectively. Furthermore, we have demonstrated that Mobius and &Cube offer a great opportunity to create new IoT applications and services in smart homes and smart offices, respectively [43,44]. Figure 5 illustrates the overall architecture for an IoT system composed of Mobius and &Cubes connected with WSN-based systems.

The Mobius provides 12 CSFs defined in the oneM2M standards. To interwork with exterior points of &Cubes, Mobius provides bindings for HTTP, MQTT and CoAP protocols. As shown in Figure 5, Mobius provides REST open APIs with which the WSN elements (e.g., sensor and actuator nodes) can be accessed via the corresponding &Cube, so that developers can create IoT applications working with any WSN systems regardless of which industry domain they belong.

For the field domain in oneM2M standards, we have developed an IoT device software platform, called &Cube. Recently, we presented details on the architecture and design principles of &Cube in our published literature [42]. In this paper, we focus on the functionalities of &Cube from a perspective of the oneM2M standards and open API.

Several variations of &Cube have been implemented for providing a base for various IoT devices, i.e., serving as MN, ASN, or ADN in the field domain. Among them, &Cube for ASN is employed in building our IoT application services, as shown in Figure 5. &Cube is designed to act as a gateway for things not having IP addresses (i.e., giving WSN elements Internet connectivity). Following the oneM2M specification, the reference points with Mobius are implemented with REST APIs, that is one of the CRUD operations followed by the URI of a target resource, along with a particular XSD (XML schema definition) format for data exchange.

However, there is no detailed implementation guideline on how we can efficiently implement the data collection or device control procedure by internally working with an IoT device. Moreover, considering that a wide variety of ‘things’ (i.e., sensors and actuators) will be incorporated into IoT devices sometimes via various wireless networks (e.g., ZigBee, WiFi, Bluetooth), we should provide developers with an efficient way of interacting with the things in a standardized form. That is, for a given thing, we need to provide a list of URIs for which its data or control variables are available. To this end, we introduce a thing-specific layer for helping developers perform the tasks described above, called a thing adaptation layer.

### 3.3. Thing Adaptation Layer

The thing adaptation layer (TAL) was first introduced in our previously-published literature [42], as an intermediate level for bridging &Cube and embedded things, e.g., sensors and actuators directly wired to IoT-based consumer electronics. In this section, we demonstrate how the TAL serves as an abstraction layer that allows the data and functions of embedded things to be exposed outside of the IoT device via corresponding REST open APIs.

**Figure 6:**
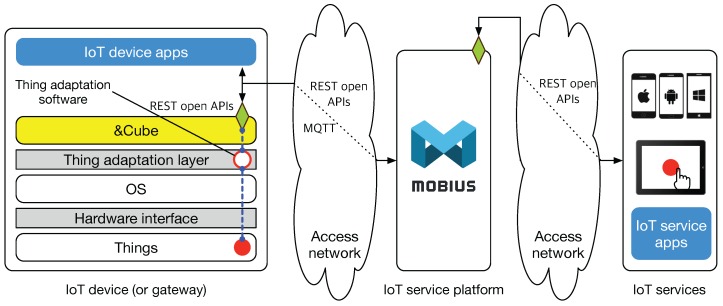
A use case for providing IoT services based on open APIs exposed through oneM2M IoT software platforms, &Cube and Mobius.

Figure 6 shows a use case for providing an IoT service based on Mobius and &Cube. The left side of the figure shows the architecture of an IoT device. As shown in the layered architecture, we assume that our target IoT device (or gateway) consists of a set of things (e.g., sensors and actuators) to provide their functions, for instance LEDs and motors for washing machines. We also assume that hardware control systems for those devices (e.g., microcontroller systems) are integrated with an operating system (OS) and supported with device drivers and relevant APIs through underlying hardware interfaces for the embedded things. With the assumptions, we now present the TAL filled with thing-specific thing adaptation software (TAS), which (e.g., the empty circle in Figure 6) is intended to support interworking between &Cube and a specific thing embedded into the device (e.g., the filled circle in Figure 6).

A TAS for a thing can be defined as a set of functions. The below code snippet shows a TAS example consisting of two functions: data collection and thing control.

// TAS for data collection
data_for_thing {
  read_data_from_thing(); // supported by the thing’s device driver and APIs
  convert_data_to_XSD();  // convert collected data into oneM2M-defined XSD
  write_XSD_to_&Cube();    // write the XSD into the resource of the &Cube
}

// TAS for thing control
control_for_thing {
  read_XSD_from_&Cube();     // read the XSD from the resource of the &Cube
  convert_XSD_to_command(); // convert the XSD into command for the thing
  send_command_to_thing();  // supported by the thing’s device driver and APIs
}
    

As demonstrated above, the main role of a TAS function is data adaptation, that is converting data collected from a given thing into the standardized format that &Cube can understand for further processing (e.g., data_for_thing). Similarly, a TAS function supports translating a command message sent from &Cube into a corresponding control command for the thing (e.g., control_for_thing). Additionally, we use the name of a TAS function (e.g., data_for_thing and control_for_thing) as the resource name for &Cube. This implies that a TAS function for a given thing will be exposed with the resource, which is created under the same name as the TAS function, and subsequently, the TAS function will be accessed with a full URL, i.e., a REST API address, combined with the base URI of &Cube and the resource name (i.e., the TAS function name).

In Figure 6, for example, the TAS function (the empty circle) for a given thing (the filled circle) is exposed with the resource name (i.e., same as the function name), which can be accessed by its REST API address (the diamond in &Cube). Under the oneM2M platform operations between the IN and MNs or ASNs, the resource of &Cube will be naturally created in Mobius with the same resource name and, in turn, can be accessed from IoT services via REST APIs. Based on the TAS-based API architecture, developers can build application software that allows users to interact with the things embedded within an IoT device.

### 3.4. From WSN towards WoT

**Figure 7:**
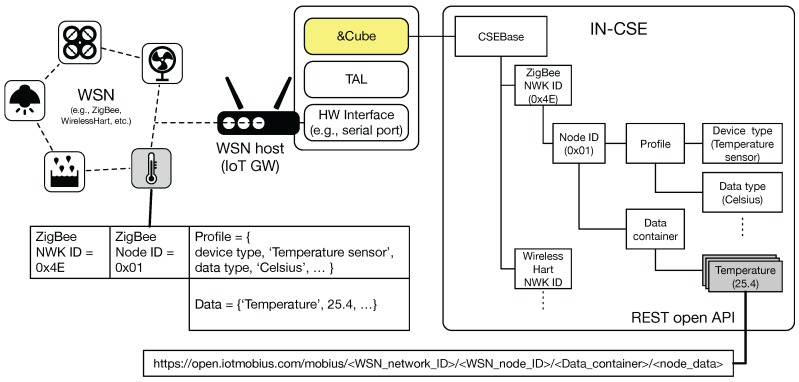
Employing oneM2M platforms (i.e., Mobius and &Cube) and TAS for extending previously-existing WSN (e.g., ZigBee, WirelessHART) systems towards the Web of Things (WoT).

In the above section, we have mainly explained that the TAS is proposed as an intermediate module converting the embedded thing-specific data to the oneM2M-defined resource model and creating REST APIs that allow access to the converted resources and, in turn, the embedded things. Similarly, TAS can be also used to extend previously-existing WSNs towards the world of WoT. Figure 7 demonstrates the way of integrating existing WSNs into the oneM2M platforms.

Here, we assume that regardless of the type of WSN protocol (e.g., ZigBee, WirelessHART or ISA100.11a), a WSN host (i.e., IoT gateway) would be wirelessly connected to the networks and collect all sensor node data through a simple hardware interface, such as a serial port. Figure 7 shows a ZigBee network example that illustrates the process of converting data of a wireless sensor node (i.e., a temperature sensor) into oneM2M resources (i.e., temperature values). The ZigBee NWK ID and node ID resources are hierarchically created under the CSEBase (e.g., https://open.iotmobius.com/mobius/). Under the ZigBee node ID resource, the profile information and collected data of the node would be mapped into different resource groups, i.e., profile and data container. Finally, as described in Section 3.1 with Figure 3, the temperature values would be able to be accessed via its corresponding REST API.

Accordingly, TAS is also considered an IPE explained in Section 3.1, which can offer interworking capability between oneM2M and non-oneM2M systems by translating each other’s different massage protocols and data models. As shown in the ZigBee-based temperature sensor node example, TAS would be responsible for translating WSN message protocols (e.g., WirelessHART or ISA100.11a) and mapping into oneM2M resource models, finally extending previously-existing WSNs into WoT. However, some aspects need to be improved according to the next release of the oneM2M standard, e.g., security token integration between two systems.

## 4. Common Open API for IoT Systems

In Section 3, we presented the oneM2M-compliant platforms and their organization, including Mobius and &Cube. Based on those platforms, we introduced our TAS-based open API architecture where a REST API for interacting with a thing embedded into an IoT device is exposed through Mobius and &Cube with the same name as the corresponding TAS function. In this section, we will provide a deeper insight into our TAS-based open API architecture to clearly show how the approach could provide end users and application developers with flexible use cases and reusable API-based development, respectively.

### 4.1. Flexibility

Service scenarios associated with IoT devices will likely occur at different scales in the IoT environment: in a localhost, on a local scale, and on a global scale. First, offline functions for a given IoT device could be conducted in a localhost. For example, when we imagine an IoT-based, WiFi-connected washing machine probably emerging in the near future, rich washing features like hand wash, wool wash, and quick wash, can naturally be created based on open APIs and performed by pressing input buttons on the control panel of the washing machine. We could also carry out the same washing tasks by manipulating a touch panel for the home automation system locally interconnected with the washing machine via a home area network. Such use cases could be performed on a local scale, i.e., within a local area network. The last scenario stands for a global-scale use case. For example, when a user wants to remotely control the washing machine from his or her workplace via a smartphone app, open APIs for globally accessing and controlling the washing machine will be clearly required to support such use scenarios. Accordingly, an efficient suite of open APIs needs to support distinct service scenarios likely occurring in the IoT environment, in particular with minimum effort from both the user and developer perspectives.

**Figure 8:**
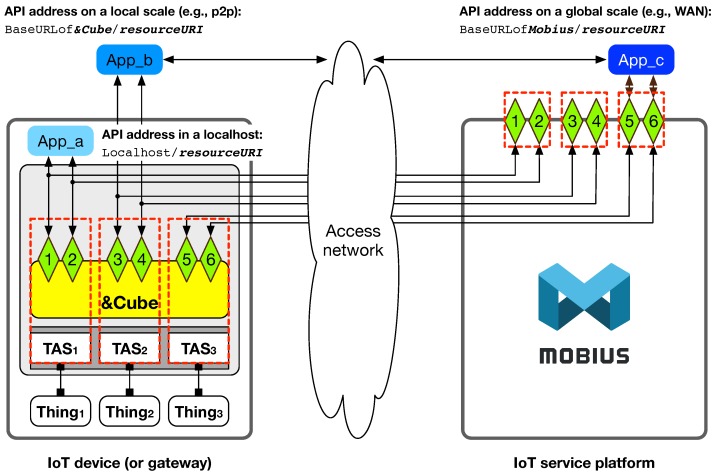
Application development support with Mobius and &Cube on several scales: localhost, local scale, and global scale.

Figure 8 shows the overall diagram of our open API architecture supporting the three service scenarios described above. In the figure, the device has three things and corresponding TAS, each of which provides application developers with a set of open APIs for the relevant thing, i.e., APIs 1 and 2 for Thing_1_, APIs 3 and 4 for Thing_2_ and APIs 5 and 6 for Thing_3_. Following the TAS-based open API architecture, each API is consistently exposed in the localhost, local area network (i.e., &Cube), and global area network (i.e., Mobius) with the same name as the corresponding TAS function. In the example shown in Figure 8, there are three application software created by developers using the six open APIs: App_a for Thing_1_, App_b for Thing_2_, and App_c for Thing_3_; they each represent the use case of the IoT device in a localhost, on a local scale, and on a global scale, respectively. As shown from the figure, we can know that by changing the base uniform resource locator (URL) setting (i.e., Localhost in the case of localhost; BaseURLof&Cube in the case of local-scale addressing; BaseURLofMobius in the case of global-scale addressing), application software can interact with the IoT devices independent of its physical location. Consequently, we can conclude with confidence that our TAS-based open API method enables developers to efficiently and rapidly create flexible application software in various working conditions for end users.

### 4.2. Reusability

Our open API-based approach can be further improved by employing uniquely identifiable IoT devices just like MAC addresses for conventional network devices. For example, provided a well-structured ID policy and resolution server, the REST open API could be used more efficiently by incorporating unique IDs for IoT devices into the API URL address. In our previously-published paper [43], we have introduced a novel ID structure based on object identifiers with which IoT devices become globally and uniquely identifiable. In this section, we first briefly explain the object identifier-based ID scheme for IoT devices and then demonstrate the nature and advantage of our common open APIs with respect to overall system organization and ecosystem establishment.

#### 4.2.1. OID-Based Identification for IoT Systems

An object identifier (OID) consists of a set of nodes, each of which is hierarchically assigned from its parent node with successive numbers separated with dots. Each node in the tree can be identified by communicating with OID repositories and the manufacturers’ OID resolution servers. Our proposed ID scheme starts with the unique root ID (e.g., 0.2.481.1 representing IoT devices in Korea) issued by a standard authority (e.g., ISO or ITU-T), followed by the manufacturer ID, model number, and serial number. For example, an OID of 0.2.481.1.1.789.7575 represents an IoT device whose manufacturer is 1 (e.g., KETI), model number is 789 (e.g., temperature sensor), and serial number is 7575.

#### 4.2.2. Common Open API

**Figure 9:**
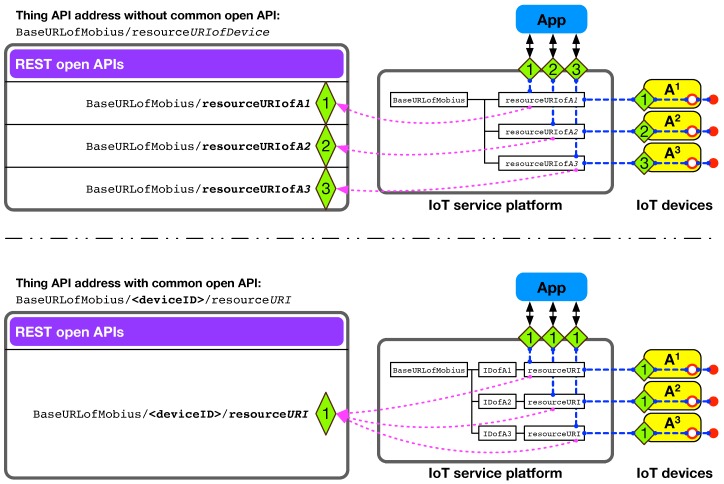
Application development support with reusable common open APIs by introducing the device ID into the resource hierarchy of the IoT service platform.

The proposed OID-based ID scheme for IoT devices can further improve our open API usability in the Mobius and &Cube-based IoT system. Figure 9 shows how the common open APIs containing device IDs can be reused when developing IoT application software. At the top of the figure, there are three IoT devices with the same type, A^1^, A^2^, and A^3^ (i.e., same model devices from the same manufacturer), each of which has a connected thing (i.e., the filled circle). In this example, however, we could not apply the TAS-based API naming convention, because the three equally-named resources (e.g., resourceURI for the circles) will obviously cause a conflict problem (i.e., three resources with the same URI) in the IoT service platform. Accordingly, we need to necessarily name them differently, e.g., resourceURIof*A1*, resourceURIof*A2* and resourceURIof*A3*, in turn producing different open API URIs, as shown at the top left of Figure 9. One way to resolve the conflict problem while holding the same resource name for three devices is to put a device ID between the base URL and common resource name, as shown in the resource hierarchy at the bottom right in Figure 9. We have used our OID-based ID scheme for the device ID, which will differentiate the equally-named resources across the same-type devices. As a consequence, the resource structure of all three devices, A^1^, A^2^, and A^3^, is able to have a common resource name (e.g., resourceURI), thus giving a common open API for interacting with the filled circle, as shown at the bottom left in Figure 9. In the example URI of the common open API for interacting with the filled circle, <deviceID> needs to be changed according to the IoT devices (i.e., A^1^, A^2^, and A^3^).

### 4.3. Comparison with Commercial APIs

In this section, we compare the proposed API with those provided by three commercial IoT platforms, including Xively [45], Nest [46], and Withings [47]. Xively is a well-known IoT platform that enables connecting and managing IoT products in such a way that people can utilize them and their data through open APIs. Nest also provides cloud-based APIs allowing one to access the connected home devices, such as a smart programmable thermostat and Internet-connected camera. Withings introduced several health monitoring devices, such as a portable blood pressure monitor, and provide APIs with which measured values are retrieved from the server. The following summarizes the full URLs provided by all of the chosen platforms in order to retrieve data from their server:

- Mobius API:
  GET https://open.iotmobius.com/mobius/<DeviceID>/<container>/<contentInstance>
- Xively API:
  GET https://api.xively.com/v2/feeds/<feed_id>/datastreams/<datastream_id>
- Nest API:
  GET https://developer-api.nest.com/devices/<DeviceType>/<DeviceID>
- Withings API:
  GET https://wbsapi.withings.net/measure?action=<Functions>&userid=<UserID>

Table 1 summarizes the comparison of Mobius API and those of the chosen commercial platforms. All of the platforms support REST APIs to access their data, and Nest also supports Firebase API for real-time synchronization, e.g., subscription and notification function. Xively and Nest use the platform- and device type-wide unique ID scheme for identifying devices, respectively. In contrast, Withings devices can be distinguished within the platform by composing the user ID and the ‘action’ query string describing a specific function supported by a target device. The user ID is supported by all platforms, except Mobius. Although its underpinning standard, oneM2M, defines a CSF for user ID management (called service charging and accounting), we have left its corresponding part for Mobius undeveloped because the CSF development is closely related to the service provider’s account management system. Mobius and Xively support flexible document formats (XML, JSON, or CSV) and underlying protocol bindings (HTTP, MQTT, or CoAP). However, Mobius is the only one that is compliant with IoT standards, such as oneM2M and OID, whereas the other platforms utilize their own proprietary systems, having a limitation on interworking between different platforms (e.g., between Xively and Nest).

## 5. IoT Service Development Using a Common Open API

This section demonstrates three IoT services built on common open APIs offered by the oneM2M platforms: Planty, iThing, and iDrone. While illustrating the example services, we will explain the full details on how the TAS-based common open APIs working with Mobius and &Cube can help application developers create innovative products and new services.

### 5.1. Planty

**Figure 10:**
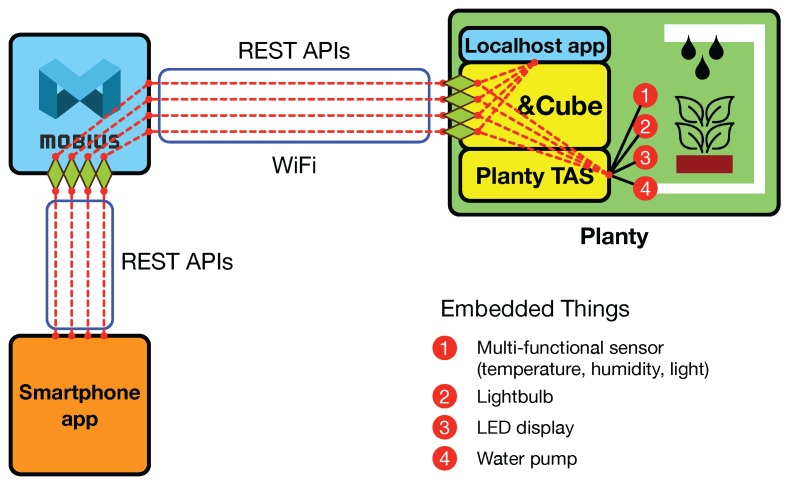
System architecture for Planty service development.

Planty is a WiFi-enabled smart flowerpot that allows users to remotely monitor and grow their plant. Figure 10 illustrates the overall diagram for the system organization of the Planty service. Planty includes an ARM Cortex 9-based hardware device, called IoTG100, consisting of a 1-GByte memory, a WiFi/Bluetooth module, a 2.4-GHz IEEE 802.15.4 ZigBee transceiver, and an SD card socket necessary for the system boot and OS image load, as shown in Figure 13a.

The IoTG100 works as an IoT gateway to the things embedded into Planty, including a multi-functional sensor for measuring temperature, humidity, and illumination, a light bulb, an LED display for showing its status, and a water pump for watering the plant (displayed with the filled circles in the right Planty box of Figure 10).

We have built four TAS functions corresponding to the embedded things’ operations as shown below:

// TAS code example for Planty
// Create four TAS functions
get_multi_functional_sensor(int option) {
   // TAS function 1 for retrieving data from the multi-functional sensor
   // (option 0: all, 1: temperature, 2: humidity, 3: illumination)
   ...
}

set_light_bulb(Boolean state) {
   // TAS function 2 for controlling the light bulb
   // (state true: on, false: off)
   ...
}

set_led_display(int option) {
   // TAS function 3 for controlling the LED display
   // (option 0: date, 1: temperature, 2: humidity, 3: illumination)
   ...
}

set_water_pump(Boolean state) {
   // TAS function 4 for controlling the water pump
   // (state true: on, false: off)
   ...
}


Each TAS function can be implemented with the library functions provided by the corresponding hardware manufacturer. For example, the TAS Function 2, set_light bulb(), would be developed with a library function setting the digital output pin of the IoTG100 connected to the light bulb to “1” to turn it on or “0” otherwise. All of the TAS functions mentioned above will be exposed at &Cube, as well as Mobius through the REST APIs (i.e., four diamonds in Figure 10), with which application developers can create a smartphone app for monitoring and controlling Planty at anytime and from anywhere. When using the REST APIs, the parameters (e.g., Boolean state) will be conveyed to the TAS functions (e.g., set_light bulb()), with them included in the HTTP request body (e.g., XML).

As we explained so far, our TAS-based common open API approach allows device manufacturers to create their IoT products independently of application developers. In other words, regardless of the hardware architecture and system organization of a given IoT device, third party application developers can create application software based on the set of open APIs for the device. As an example, Table 2 illustrates the comparison of API URLs for controlling the water pump installed into Planty. The TAS function name is given by set_water_pump, and all of the REST APIs commonly share the name in the full URL addresses. Accordingly, when developers create a watering function by pushing a button on Planty (i.e., working in a localhost), the REST API required will be POST https://localhost/ncube/set_water_pump. In contrast, when developers build a smartphone application working globally, the REST API will be POST https://open.iotmobius.com/mobius/<deviceID>/set_water_pump, where <deviceID>, e.g., 0.2.481.1.1.790.0001 is plugged in. Note that the single URI (same as the corresponding TAS function name) is shared among the REST APIs for controlling the water pump in localhost, a local area, and a global area. It should be also noted that all Planty products manufactured will be able to share a single REST API for accessing them in a global area by replacing the deviceID with the target Planty’s OID, whereas Xively creates duplicate APIs for the exact same type of device registered. Figure 13d shows a capture image of the Planty service, and the full demo video is available at YouTube [48].

### 5.2. iThing

**Figure 11:**
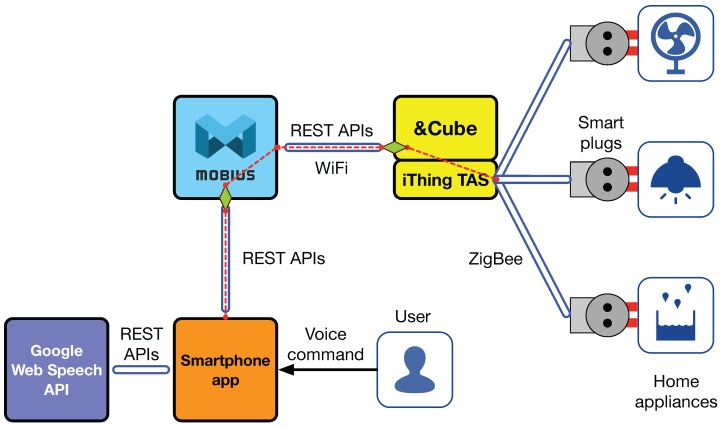
System architecture for iThing service development.

The iThing service provides a voice-based home appliance control for smart homes. To control the electricity power consumption of existing home appliances, we have developed smart plugs consisting of a current sensor, a power relay, and a ZigBee wireless transceiver, as shown in Figure 13b. We described the detailed design and implementation of the smart plug in our previous literature [49]. The smart plug can be used as both a sensor and actuator. That is, it can measure electrical power used in a home appliance plugged in and also instantly turn it on or off by sending a command signal through a ZigBee-based wireless network, as shown in Figure 11.

We have developed two TAS functions for measuring and controlling the electricity power consumption, though the power control TAS function is only employed in the iThing service:

// TAS code example for iThing
// Create two TAS functions
get_smart_plug(int index) {
   // TAS function 1 for retrieving electricity consumption data from the smart plugs
   // (index 0: fan, 1: light bulb, 2: humidifier)
   ...
}

set_smart_plug(int index, Boolean state) {
   // TAS function 2 for controlling the smart plugs
   // (index 0: fan, 1: light bulb, 2: humidifier)
   // (state true: on, false: off)
   ...
}


Similar to the previous example, the TAS functions are exposed at both &Cube and Mobius through the REST open API with which application developers can create smartphone applications to control the electricity power of home appliances.

Especially, the iThing smartphone app leverages the voice recognition feature to provide users with an easy-to-use user interface. Instead of developing our own machine learning algorithm for voice recognition, we have used Android’s SpeechRecognizer API (i.e., another set of open APIs) for converting a user’s verbal command to text via Android phones. According to the interpretation of the user’s command, Mobius will send appropriate control signals (i.e., on/off) to smart plugs, i.e., home appliances. The following code example shows our implementation of using Android’s SpeechRecognizer API for voice recognition applications:


// Android app code example for iThing
// Create and start Android RecognizerIntent for voice recognition
Intent intent = new Intent(RecognizerIntent.ACTION_RECOGNIZE_SPEECH);
intent.putExtra(RecognizerIntent.EXTRA_LANGUAGE_MODEL, “en-US”);
startActivityForResult(intent, RESULT_SPEECH);

// When RecognizerIntent activity receives voice recognition result,
// the following function will be overridden
@Override
onActivityResult(int requestCode, int resultCode, Intent data) {
   switch(requestCode) {
   case RESULT_SPEECH:
   if (resultCode == RESULT_OK && data != null) {
      // check the result status from RecognizerIntent’s result code
	  // get recognized text from Android RecognizerIntent
	  ArrayList<String> text = data.getStringArrayListExtra(RecognizerIntent.EXTRA_RESULTS);
	 
	  // find request functions (Mobius API) from recognized text
	  findRequestFunctionMap(ArrayList<String> text);
   }
   break;
   }
}

findRequestFunctionMap(ArryList<String> text) {
   String functionName = text.toString();
   switch(functionName) {
   // Here, we assume that ‘fan,on’ is a sequence of reserved words
   // comma-separated to turn a fan on
   case “fan,on”:
   // To turn the fan on (i.e., set the smart plug to ‘1’),
   // we can use the API registered above as a TAS function,
   // set_smart_plug(int index, Boolean state) as follows:
   // POST https://open.iotmobius.com/mobius/0.2.481.1.1.791.0001/set_smart_plug
   // with the XML body describing index = 0, state = true
   ...
   }
}


The iThing service development shows a good example of the usage of our common open API. Recall that Xively creates different APIs for the same type of devices registered as Feeds. In contrast, three same-type IoT devices (i.e., smart plugs) can share a single common open API for controlling power consumption (i.e., the diamond), and smartphone apps will use the API with three different device IDs; in our case, three OIDs having the same manufacturer and model number, but different serial numbers. Figure 13e shows a capture image of the iThing service, and the full demo video is available at Youtube [50].

### 5.3. iDrone

Our last example service is the iDrone, a connected drone (i.e., unmanned aerial vehicle) that we can control in (almost) real time. As demonstrated in the Consumer Electronics Show (CES) this year, a variety of drone-based services (e.g., delivery, photography and video, entertainment, first aid, and military operation) will be one of the prominent application areas in IoT industry verticals.

**Figure 12:**
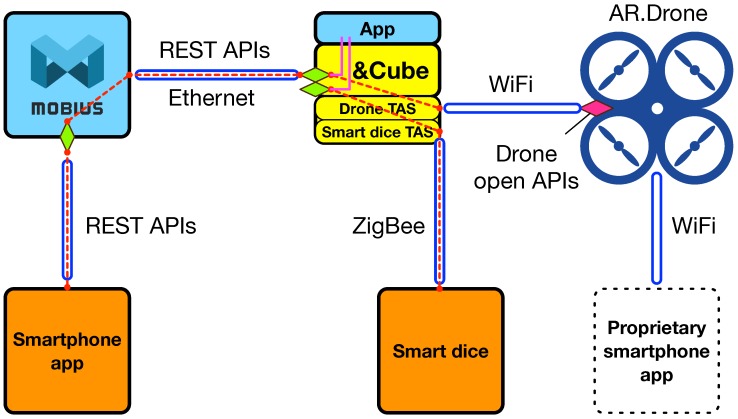
System architecture for the iDrone service development.

Considering such a hot trend of integrating IoT with drones, we have developed a smartphone app that sends control commands to a Parrot AR.Drone using its open APIs and our platforms. There already exists a proprietary smartphone application software, called AR.Freeflight, created by the company Parrot, providing original full functions including movement control, GPS-based navigation, image capture, and video recording. However, it also has launched the AR.Drone open API game development platform for third party developers to create their new innovative applications working with AR.Drones [51]. Accordingly, using the AR.Drone SDK (software development kit), we have designed a system architecture for the iDrone service as shown in Figure 12.

In order to incorporate the AR.Drone with &Cube, we have first developed the Drone TAS (the upper one of two diamonds in &Cube), designed to map the input commands from the smartphone app to the appropriate Drone open APIs provided (displayed as a diamond next to the AR.Drone). With the APIs, the AR.Drone can be controlled by sending text strings, called *AT Commands*. One AT command consists of a format string of AT*‘command name’=‘sequence number’,‘a list of comma-separated arguments depending on the command’<CR> where a CR (carriage return) character stands for the end of the AT command. A set of commands are defined with their corresponding command names, for example AT*REF for takeoff or landing and AT*PCMD for moving.

// TAS code example for iDrone
// Establish a UDP connection with an AR.Drone
UDPbuffer udp_buffer = connect_ar_drone();

// Create a take-off TAS function using a format string starting with AT*REF command
ar_drone_takeoff() {
   // AT command format for take-off
   // AT*REF=%d,%d<CR>
   // Argument1: the sequence number
   // Argument2: an integer value representing a 32 bit-wide bit-field controlling the drone
   // - Bit 9 is used in the control bit-field for take-off/landing
   // - Bit 8 is used in the control bit-field for emergency stop/reset
   // - Bits 18, 20, 22, 24 and 28 should be set to 1
   // - Other bits should be set to 0
   
   // e.g., AT*REF=1,22282752<CR>
   // Here, 22282752 is an integer representing a 32 bit-wide bit-field of
   // ‘0000 0001 0101 0100 0000 0010 0000 0000’
   
   string AT_command = “AT*REF=1,22282752\r”;
   sprintf (udp_buffer, AT_command);
}

// We can create a list of TAS functions for controlling AR.Drones,
// each of which is configured to send appropriate AT commands
ar_drone_landing();
ar_drone_move_stop();
ar_drone_move_front();
ar_drone_move_back();
ar_drone_move_left();
ar_drone_move_right();
ar_drone_move_up();
ar_drone_move_down();
...


Currently, we have implemented the Drone TAS for performing basic controls (e.g., take-off, landing, forward, backward, left, right) and flying a predefined path. However, by building a well-customized TAS function (i.e., an AT command configured to perform a specific drone moving), it could provide a rich set of commands or perform more complicated tasks, such as autonomous navigation.

**Figure 13:**
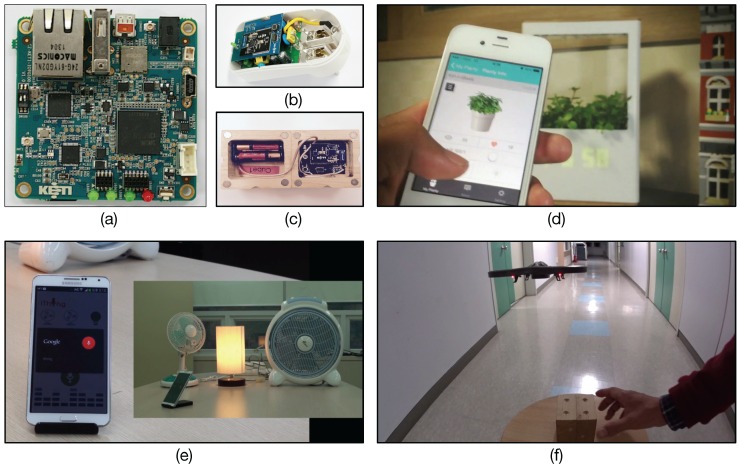
The prototype IoT devices and services: (**a**) IoT gateway IoTG100, (**b**) smart plug (**c**) smart dice, (**d**) Planty, (**e**) iThing and (**f**) iDrone.

The smartphone app and Mobius-based drone control sometimes cause a latency issue depending on network traffic or server response time. Moreover, drone control without visual feedback might not be suitable for real-time service delivery. As a result, we have developed the smart dice embedded with a three-axis accelerometer and ZigBee transceiver, as shown in Figure 13c. The smart dice provide six different inputs by positioning each sensitive axis (X, Y, Z) of the accelerometer at +g and −g, each of which will be converted to a specific command as defined in the Drone TAS explained above to perform a task. To this end, we have implemented a TAS function for the smart dice (the lower one of two diamonds in &Cube):

// TAS code example for iDrone (smart dice)
// Create a TAS function to obtain the current side of the dice
// (side: 1, 2, 3, 4, 5, 6)
get_which_side_up(int side) {
   // Retrieve 3-axis acceleration values
   read_accelerometer(int x, int y, int z);
   
   // Decide which side comes up
   decide_which_side_up() {
   // Create an algorithm to calculate which side comes up
   // using x, y, z-axis acceleration values
   ...
   
   side = algorithm_result;
   }
}


Finally, we have created a device app on &Cube, as shown in Figure 12, which utilizes the smart dice TAS in order to decide which side comes up, and its result will call a corresponding AR.Drone TAS function to move the drone accordingly.

This smart dice-based drone control will be employed in a local area network, and thus, does work in almost real time. Furthermore, both applications for drone control present the flexible usability of our TAS-based open API method in IoT service development collaborating with other vendors’ open APIs well. Figure 13f shows a capture image of the iDrone service, and the full demo video is available at YouTube [52].

## 6. Limitations and Remaining Challenges

A myriad of WSN systems being non-compliant with oneM2M standards have been deployed and are working well in a wide array of industry verticals. Indeed, most of those systems rely on their own proprietary platforms, protocols, or standards. Such previously-existing legacy systems will be able to be easily integrated with IoT solutions by developing the corresponding appropriate TAS (i.e., oneM2M IPE) modules in the oneM2M platforms. However, the oneM2M global initiative has also noticed the importance of interworking with other M2M/IoT standards and collaborated with company alliances, such as AllSeen Alliance and Open Interconnect Consortium (OIC), to achieve a global interoperability between oneM2M and their standard platforms. In this circumstance, we will continue to investigate the interworking solutions with their open source framework, i.e., AllJoyn and IoTivity.

WSN systems rely on a wide variety of heterogeneous hardware systems with respect to form factor, power source, network, sensor, actuator, computing processor, etc. In particular, in the case of WSN systems having constrained resources (e.g., ultra-low power, wireless sensor nodes) designed to work in constrained environments (e.g., low bandwidth network connection), their Internet connectivity will be a key to reaching the global IoT infrastructure. Accordingly, the oneM2M initiative has been working on establishing standardized application protocols that can be bound to HTTP, MQTT, and CoAP to support such Internet connectivity in constrained REST environments. Currently, &Cube connects with oneM2M service platforms (e.g., Mobius) via HTTP and MQTT protocols, but not yet CoAP. Furthermore, &Cube is designed to run on the Java runtime environment (JRE), and thus, its system resource requirements could not be supported by the WSN elements with constrained resources. Thus, we plan to build another version of &Cube based on native programming languages, which supports the CoAP protocol and constrained resource architecture specified in the oneM2M specification.

Although the OID-based device ID structure has a significant advantage in building IoT solutions, several practical questions may need to be addressed. First, we must consider which standard authorities will be responsible to issue and manage the manufacturer nodes for the OID tree. Since the OID-based ID scheme should be globally identifiable, a group of international standards bodies need to systematically manage the ID issuing process and constantly manage the failures of all OID resolution servers. We also may consider a federation of OID resolution servers for SMEs (small and medium-sized enterprises), because they would not be able to run independently due to a high operating expenditure (OPEX). Second, our common open API scheme is designed to set OID-based IDs inside REST interfaces as shown Section 4.2.2. Here, a full URL address may be too long and inefficient to deal with, in particular in case of resource-constrained embedded systems. One method to avoid this long URI issue is to make use of pseudonym URIs composed of a random number and text just like Google’s youtu.be URI shortener. Thus, the pseudonym URI method can provide APIs with a shortened URI, but also hide the resource topology in IoT platforms, resulting in increasing security. All of the identification schemes we have mentioned require a process of standardization, involving a wide array of players across multiple industries. Accordingly, we have been working on activities to standardize the scheme and architecture for IoT devices and services, in particular actively participating in oneM2M working groups. The OID-based ID scheme and pseudonym URI method are currently included in the oneM2M specification document [32].

## 7. Conclusion

For the past few decades, wireless sensor network (WSN) systems have been at the core of building service applications in a wide range of industry verticals, including home, building, power grid utilities, transportation infrastructure, factory, healthcare, etc. However, as the recent technological revolution called the Internet of Things (IoT) attracts more and more attention, a standardized method is required for integrating previously-existing as well as future WSN systems into the IoT-enabled solutions.

In this paper, we have presented full details of how standard IoT platforms and common open APIs can help existing and future WSN systems evolve out of just local sensing and actuating parts into IoT solutions with a standardized Internet connectivity incorporated. Through an extensive investigation on the oneM2M standards, we have developed Mobius, which works as a server platform for IoT services. We have also developed &Cube, which works as an IoT gateway to WSN elements with no access to the Internet. These platforms are designed to provide REST application programming interfaces (APIs) for interacting with the things equipped into a given WSN system in various scales: in a localhost, in a local area network, and in a global area network. Furthermore, the REST API structure employs the globally-identifiable device ID scheme based on object identifiers (OIDs), allowing open APIs to be commonly shared between the same-type devices, and thus, increasing the reusability of the common open APIs in developing IoT services. With the pilot IoT service development, we have demonstrated the advantages and practical usability of our IoT platforms and common open APIs. We will continue to work on widespread adoption of our platforms and open API architecture in a variety of IoT systems through standardization efforts, as well as interworking proxy development between heterogeneous IoT platforms.

## Figures and Tables

**Table 1:** Comparison of Mobius API and commercial APIs provided by Xively, Nest, and Withings.

Platform	API type	Device ID	User ID	Body	Protocol	Standards
Mobius	REST	◯ (globally-unique)	×	XML, JSON	HTTP, MQTT, CoAP	oneM2M, OID
Xively	REST	◯ (platform-wide unique)	△ (included in header) as an access token)	XML, JSON, CSV	HTTP, MQTT	×
Nest	REST, Firebase	◯ (device type-wide unique)	△ (included in header) as an access token)	JSON	HTTP	×
Withings	REST	× (identified with user ID and action)	◯ (included in header) as an access token)	JSON	HTTP	×

**Table 2:** Given a thing adaptation software (TAS) function (i.e., set_water_pump), for the water pump in Planty, a comparison of corresponding REST API URLs for controlling it: in localhost, in a local area network, and in a global area network.

API scope	Method	API URLs
Localhost	POST	https://*localhost*/*ncube${}^{a}$*/set_water_pump
Local area	POST	https://*192.168.0.1*/*ncube${}^{a}$*/set_water_pump
Global area	POST	https://*open.iotmobius.com*/*mobius${}^{a}$*/
		*0.2.481.1.1.790.0001${}^{b}$*/set_water_pump

*^a^* We assume the localhost/ncube, 192.168.0.1/ncube, open.iotmobius.com/mobius are the base URLs of the three examples. The local IP address of Planty is given by 192.168.0.1. *^b^* In the example, we assume the device ID of Planty is given by 0.2.481.1.1.790.0001.
